# Alternative Splicing Events and Their Clinical Significance in Colorectal Cancer: Targeted Therapeutic Opportunities

**DOI:** 10.3390/cancers15153999

**Published:** 2023-08-07

**Authors:** Mosebo Armstrong Manabile, Rodney Hull, Richard Khanyile, Thulo Molefi, Botle Precious Damane, Nigel Patrick Mongan, David Owen Bates, Zodwa Dlamini

**Affiliations:** 1 SAMRC Precision Oncology Research Unit (PORU), DSI/NRF SARChI Chair in Precision Oncology and Cancer Prevention (POCP), Pan African Cancer Research Institute (PACRI), University of Pretoria, Pretoria 0028, South Africa; u20804289@tuks.co.za (M.A.M.); rodney.hull@up.ac.za (R.H.); richard.khanyile@up.ac.za (R.K.); thulo.molefi@up.ac.za (T.M.); david.bates@nottingham.ac.uk (D.O.B.); 2 Department of Medical Oncology, Faculty of Health Sciences, Steve Biko Academic Hospital, University of Pretoria, Pretoria 0028, South Africa; 3 Department of Surgery, Steve Biko Academic Hospital, University of Pretoria, Pretoria 0028, South Africa; precious.setlai@up.ac.za; 4 School of Veterinary Medicine and Science, University of Nottingham, Nottingham NG7 2QL, UK; nigel.mongan@nottingham.ac.uk; 5 Centre for Cancer Sciences, Division of Cancer and Stem Cells, Biodiscovery Institute, University of Nottingham, Nottingham NG7 2RD, UK

**Keywords:** colorectal cancer, alternative splicing, protein arginine methyltransferases, splicing factor kinases, AS events, splicing disrupter drugs, PRMT, PRMT isoforms, PRMT inhibitors

## Abstract

**Simple Summary:**

Colorectal cancer is the second leading cause of cancer-related deaths worldwide. The incidence of this cancer continues to rise, especially in developing countries. Alternative splicing is a normal cellular process that results in the generation of proteins with different structures and functions from a single gene. Colorectal cancer can cause dysregulation of alternative splicing processes to promote its development and growth until it spreads. Dysregulated alternative splicing processes have been shown to promote cancer survival by producing proteins that activate genes known to promote cancer development or deactivate those that inhibit cancer development. It is therefore important that dysregulated alternative splicing genes in colorectal cancer are identified for diagnosis and development of treatments that can specifically target these genes in order to stop them from promoting cancer development and progression.

**Abstract:**

Colorectal cancer (CRC) ranks as one of the top causes of cancer mortality worldwide and its incidence is on the rise, particularly in low-middle-income countries (LMICs). There are several factors that contribute to the development and progression of CRC. Alternative splicing (AS) was found to be one of the molecular mechanisms underlying the development and progression of CRC. With the advent of genome/transcriptome sequencing and large patient databases, the broad role of aberrant AS in cancer development and progression has become clear. AS affects cancer initiation, proliferation, invasion, and migration. These splicing changes activate oncogenes or deactivate tumor suppressor genes by producing altered amounts of normally functional or new proteins with different, even opposing, functions. Thus, identifying and characterizing CRC-specific alternative splicing events and variants might help in designing new therapeutic splicing disrupter drugs. CRC-specific splicing events can be used as diagnostic and prognostic biomarkers. In this review, alternatively spliced events and their role in CRC development will be discussed. The paper also reviews recent research on alternatively spliced events that might be exploited as prognostic, diagnostic, and targeted therapeutic indicators. Of particular interest is the targeting of protein arginine methyltransferase (PMRT) isoforms for the development of new treatments and diagnostic tools. The potential challenges and limitations in translating these discoveries into clinical practice will also be addressed.

## 1. Introduction

**Figure 1:**
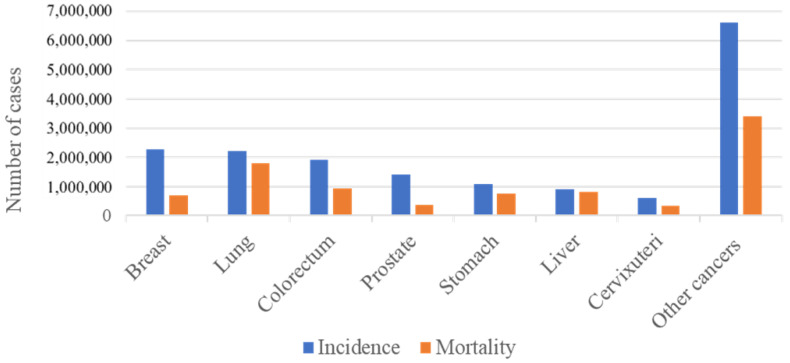
Incidence and mortality numbers for the most prevalent cancers. The figure shows the mortality and incidence numbers of the most prevalent cancers worldwide in the year 2020. Incidence reflects the number of newly diagnosed cases, while mortality reflects the number of deaths related to each cancer. It can be seen that worldwide colorectal cancer is the third most prevalent and accounts for the second most cancer-related deaths.

Colorectal cancer (CRC) has been shown to greatly contribute to mortality, morbidity, and the economic costs of healthcare worldwide. The global burden of the disease is reflected by the reported incidence of the disease being 1.8 million cases, with 0.9 million deaths, and 19 million disability-adjusted life years (DALYs) worldwide [1,2]. According to the GLOBACAN 2020 cancer statistics, CRC is the third- and second-ranked cancer for its overall incidence and mortality worldwide, respectively (Figure 1) [3].

Furthermore, the incidence of CRC is believed to be on the rise, particularly in low-middle income countries (LMICs) and Sub-Saharan Africa (SSA), which is often associated with socio-economic transitions. Its incidence has been reported to be stabilizing or decreasing in middle-high and high-income populations [1,2,4]. Given the high costs for the screening and treatment of CRC, identifying novel biomarkers for the prediction and therapeutic interventions is urgently needed. Cancer cells arise from the accumulation of several mutations in response to a variety of factors, including epigenetic alterations. Genomic instability promotes the progression from precancerous lesions to carcinoma. The commonly known genomic instability in CRC involves microsatellite instability (MSI), chromosomal instability, and chromosome translocations [1,2]. Cancer cells with genetic alterations have the ability to evade the immune system [3]. These include MSI-high cancers that can avoid recognition by the immune system by undergoing frequent immunoediting resulting in alterations in the major histocompatibility complex (MHC)-antigen presentation pathway [4].

Precursor mRNA (pre-mRNA) splicing is an important post-transcriptional process that occurs in mammalian cells. In this process, introns are removed by an enzyme complex referred to as the spliceosome, and exons are joined back together. This results in a mature mRNA ready for translation into a protein [5]. Several mRNA variants can be formed from a single gene through a process known as alternative splicing (AS). Here, introns are removed and several exons are joined together in different combinations to produce mRNA variants with equal chances to be translated into unique proteins with different, or even opposing, functions [6]. Several studies have reported that about 90–95% of mammalian genes undergo AS and are often associated with cellular homeostasis, differentiation and lineage determination, tissue growth and maintenance, and organ development [5,7]. The genetic and epigenetic alterations in molecules associated with mRNA splicing may result in the generation of aberrant mRNA transcripts which may contribute to tumorigenesis [8].

The key role of alternatively splicing events in tumorigenesis, cancer progression, and resistance to therapy has been widely recognized [9]. Therefore, understanding the contribution of alternatively spliced events in tumorigenesis and metastasis holds the potential for the development of splicing disrupter drugs as a new class of therapeutic agents [5,7,10,11]. Using high throughput technologies such as next-generation sequencing (NGS), several alternatively spliced variants have been identified with the potential to serve as prognostic and diagnostic biomarkers. These biomarkers include pre-mRNA splicing regulators such as protein arginine methyltransferases (PRMTs) and splicing factor kinases (SFKs), which are thought to play a role in the development and/or progression of different cancers. However, in colorectal cancer, alternatively spliced variants of these splicing factors and how they contribute to the development and progression of CRC in patients remains understudied [9,10,11]. Thus, in this review, we discuss the currently available knowledge on the use of alternatively spliced variants as biomarkers that are associated with the development and progression of CRC. These biomarkers hold the potential to replace and/or to be used together with the currently available screening/diagnostic methods. We further discuss the current advances in identifying alternatively spliced variants that have therapeutic potential for CRC.

## 2. Epidemiology in High-Income Countries Versus Sub-Saharan Africa

**Figure 2:**
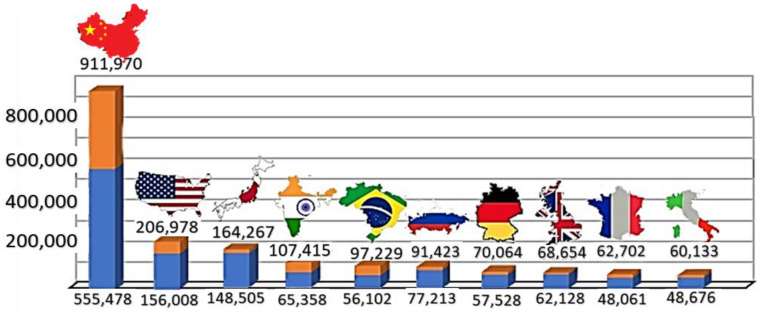
The projected increase in colorectal cancer cases by 2040. The projected increase in the number of colorectal cancer cases by 2040 in the 10 countries with the highest burden of CRC is represented by the orange bars above the blue bars, which indicate the number of new cases recorded in 2020.

The global burden of CRC presents major challenges to the world’s healthcare systems. Studies that use mathematical models to estimate the future trends and projections of CRC indicate that there will be a significant increase in new cases from the year 2020 to 2040 in 10 countries with the highest incidence and mortality rates, as indicated in Figure 2 [5,6]. The highest incidence of CRC is seen in China and is projected to increase at an alarming rate of 64% from 0.56 million in 2020 to 0.91 million in 2040, followed by the United States with 0.16 million estimated new CRC cases in 2020 to 0.21 million in 2040 [6].

Although long-term projections suggest a significant increase in new cases of CRC, currently, the high incidence and mortality rates are reported to have stabilized or decreased in several countries which fall within the high or very high human development index (HDI). These include the USA, Australia, New Zealand, and countries within Western Europe [7,8]. As a result, the overall survival (OS) rates have increased or are increasing within these countries, with patients being reported to survive longer, reaching ages between 70–75 [5,9]. Advances in the development of various treatment options as well as population-screening methods have also led to an increase/prolonged OS and this was achieved through understanding the pathophysiology of CRC [10,11].

In contrast to the stabilized or reduced incidence of CRC in Western regions, in African countries, evidence suggests that the CRC burden is on the rise, most particularly in LMICs [2,4,12,13,14,15,16,17,18]. The increase in the incidence of CRC in the LMICs is associated with socio-economic status, with more than 50% of all CRC cases attributed to poor lifestyle choices (alcohol abuse, smoking, and lack of physical activity) and the aging population [19]. Africa was reported to have an estimated population of 1.3 billion in 2018, making the African continent the second most populated continent in the world [18].

Despite the numbers currently reported, the true numbers of CRC cases and deaths related to the disease are largely unknown, with the currently available data derived from descriptive studies done in a few African countries. This is partly due to a lack of sufficient cancer registries, which are crucial in providing important data on the incidence, prevalence, and mortality rates of all cancers from the African continent [20]. Besides the paucity of data, the current and available data suggest that CRC is the fifth most common type of cancer on the African continent and the rates of CRC from SSA are lower than that of Northern Africa and much lower than high-income countries [20]. Due to insufficient data collection systems, these findings may misrepresent the actual burden of the disease. Furthermore, the lack of actual data on CRC means that it might be difficult for governments to invest in CRC management strategies.

## 3. Alternative Splicing as a Highly Regulated Process

**Figure 3:**
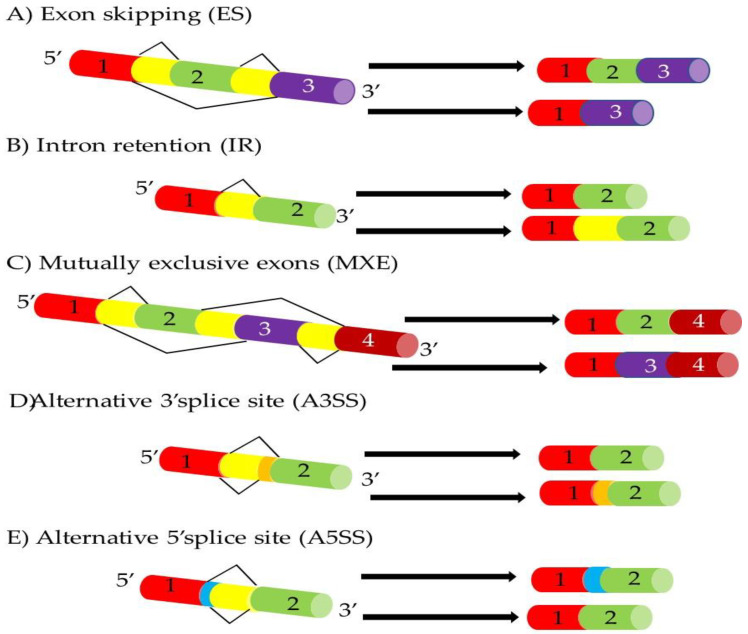
Different types of AS events. There are five main types of splicing events. In (**A**) ES (exon skipping), one or more exons that are normally included are skipped to create a truncated mRNA and protein. In (**B**) IR (intron retention), introns that are normally excised are retained to create a longer transcript and protein. In (**C**) MXE (mutually exclusive exon), not all exons are included in a single transcript, with the inclusion of one resulting in the exclusion of another. In (**D**) A3SS (alternative 3′ splice site), an alternate splice site upstream of the initial site results in the formation of a longer exon; and (**E**) A5SS (alternative 5′ splice site).

Pre-mRNA splicing is a vital stage in a variety of processes, including cellular growth, differentiation, and development of illnesses. Alternative splicing is a tightly regulated process by which different combinations of exons within a pre-mRNA molecule are spliced together, resulting in the generation of multiple mRNA isoforms [5]. This process is mediated by the spliceosome, a large and dynamic piece of molecular machinery composed of RNA and protein components [7]. The spliceosome recognizes splicing signals within the pre-mRNA sequence, such as exon–intron boundaries and splicing enhancer or silencer elements, to orchestrate the inclusion or exclusion of exons during splicing [21]. Alternative splicing is subject to intricate regulation, allowing precise control over gene expression and protein diversity [5,7,9,21]. According to data from whole transcriptome sequencing, AS is a very common event that affects about 90–95% of all expressed human genes [11]. Various factors influence AS, including tissue-specific splicing factors, RNA-binding proteins, and regulatory elements within the pre-mRNA sequence. Additionally, epigenetic modifications, such as methylation, can modulate splicing patterns by influencing the accessibility of the spliceosome to specific exons or splice sites. To date, AS events have been classified into five different types (Figure 3) that are regulated by cis-acting and trans-acting elements [6].

One of the remarkable features of AS is its tissue-specific nature [22]. Different tissues and cell types possess distinct repertoires of splicing factors, resulting in tissue-specific splicing patterns [7]. This tissue-specific AS allows for the generation of unique protein isoforms tailored to specific cellular functions and developmental stages [23]. Dysregulation of alternative splicing is increasingly recognized as a critical factor in human diseases [21]. Numerous genetic disorders and cancers have been linked to aberrant splicing events that result in the production of non-functional or disease-promoting protein isoforms [7]. While dysregulated alternative splicing is observed in cancer and can contribute to its development and progression, it is not accurate to state that alternative splicing events are solely driver events or solely consequences of carcinogenesis. The relationship between alternative splicing and cancer is complex and multifaceted, with both cause and effect interactions occurring. Further research is needed to fully understand the role of alternative splicing in cancer biology and to explore its potential as a therapeutic target.

## 4. Clinical Significance of AS Events in Cancer

There is growing interest in post-transcriptional splicing factor mutations and their roles in carcinogenesis [9]. Cancers develop as a result of alterations in gene expression and post-transcriptional modifications (PTM). This includes genetic mutations, epigenetic modifications, aberrant alternative splicing (AS), and changes in the transcription of non-coding RNAs such as miRNA. These changes can occur in response to a wide range of factors (environmental and infectious) [21]. In recent years, alternatively spliced events were associated with numerous types of cancer through the development of high throughput technologies. Interestingly, some AS events hold the potential to be explored further and used in the pre-clinical and clinical settings. A study that compared esophageal squamous cell carcinoma (ESCC) tissues to normal tissues found that a total of 45,439 AS events take place in esophageal squamous cells. The study reported that 6019 of the AS events differ significantly in ESCC tissues compared to normal tissues, resulting in differently spliced mRNA and protein isoforms unique to the disease [24]. The study further demonstrated that the splicing factor 3b subunit 4 (SF3B4) was responsible for 102 abnormal AS events in 92 targeted genes. The expression of SF3B4 was associated with survival-related genes in ESCC.

These findings were supported by other studies indicating that heterozygosity for SF3B4 mutations leads to defects in mRNA splicing, particularly exon skipping. Overexpression of SF3B4 in cancer cells also caused mis-splicing of Kruppel-like factor 4 (KLF4), a tumor suppressor-encoding gene, resulting in a non-functional transcript, and therefore promoting carcinogenesis in hepatocellular carcinoma [25,26]. Studies have also identified splicing events specific to CRC. A study by Xiong Y et al., 2018 reported that 34,334 AS events from 8942 genes were identified in CRC tissues. This means that one gene might have almost four AS events on average. Furthermore, the study showed that out of the 34,334 identified AS events, 421 AS events were differentially expressed between samples when they were divided based on clinical features such as age, sex, and OS, as well as tumor size, lymph node status, and metastasis (TNM) stage. However, the differentially expressed AS events were not compared to those expressed in normal tissue. Besides the above-mentioned studies, there are others that further indicate the important role played by dysregulated AS events in the genes associated with the development and progression of various cancer types, as shown in Table 1.

## 5. Alternative Splicing (AS) Events in Colorectal Cancer Pathogenesis

The structurally and functionally different proteins that can result from pre-mRNA splicing contribute to genetic diversity in eukaryotic cells [35]. Impaired cellular homeostasis, a major contributor to cancer, is considered to be directly related to aberrant alternatively spliced transcripts. Mutations and changes in the concentration of splice factors may contribute to cancer because alternative splicing governs the production of spliced variants and plays a crucial role in post-transcriptional regulation [36,37]. Through the occurrence of the alternative splicing event such as exon skipping, intron retention, and the choice of alternative splice sites, cancer-specific transcripts, and isoforms are produced which further impact cancer biological processes which include angiogenesis, apoptosis, cell-cycle regulation, metastasis, proliferation and invasion [37,38]. Just as in other types of cancers, AS is a hallmark in the development and progression of CRC. There are aberrant AS events that are reported to be closely associated with CRC progression.

### 5.1. Implications of lncRNAs AS Events in Colorectal Cancer

Long non-coding RNAs (lncRNAs) have emerged as key regulators in cancer biology, including CRC [39]. To date, a lot of evidence has revealed that long non-coding RNA (lncRNA) molecules are aberrantly expressed in CRC tissues or cells, which regulate gene expression and participate in the occurrence and development of CRC by regulating cell proliferation, cell cycle, epithelial–mesenchymal transition (EMT), drug resistance, and metastasis [40]. Some of the examples of lncRNAs that are aberrantly expressed and play a pivotal role in CRC carcinogenesis includes LncRNA-SNHG11, LncRNA-RPPH1, LINC01106, lncRNA-APC1, and lncRNA-AK028845 [40]. LncRNAs are also known to directly affect the function of micro-RNAs (miRNAs). Micro-RNAs (miRNAs) are part of the non-coding RNA (ncRNA) family, and miRNAs are smaller transcripts that are 18–22 base pairs long [41]. miRNAs are one of the small molecules known to regulate biological processes via the splicing of mRNA to generate alternate transcripts. For example, alternative transcripts such as miR-583-3p and miR-1273-3p were previously associated with cell growth and proliferation in colon cancers [42]. High expression of miR-340-5b is reported to promote invasion, metabolism, and EMT in CRC through the activation of the ERK signaling pathway [39,42]. There is a significant increase in the number of novel lncRNAs associated with CRC. LINC00662 is a lncRNA which plays a crucial role in colon cancer progression through the activation of the ERK signaling pathway [39,40]. Another lncRNA of interest is the colon cancer-associated transcript 1 (CCAT1), which was discovered by Nissan et al. CCAT1 has been shown to be overexpressed in various cancer types, including CRC [43]. Recent research suggests that CCAT1 promotes colon cancer cell growth by increasing expression of the oncoprotein c-MYC and the oncogenic mRNA tumor suppressor candidate 3 (TUSC3), the target of miR-181b-5p in CRC cells, thus increasing glucose metabolism to fuel colon cancer cell growth. This promotes colon cancer cell migration and invasion by accelerating the EMT process and negatively modulating miR-218 and hsa-miR-4679; and suppresses apoptosis [40]. Several other miRNAs are reported to be dysregulated in colon cancers, including but not limited to hsa-miR-585-3p, hsa-miR-1273, hsa-miR-340-5p, hsa-miR-374b-5p, and hsa-miR-335-5p [34]. Understanding the intricate network of alternative splicing in lncRNAs and its impact on CRC pathogenesis holds great promise for the development of novel diagnostic and therapeutic approaches. Targeting specific alternatively spliced lncRNA isoforms or modulating splicing factors could offer potential strategies for precision medicine in colorectal cancer.

### 5.2. Other AS Variants Associated with Colorectal Cancer

As mentioned above, AS does not occur independently. Instead, this procedure is linked to other cellular mechanisms that are frequently manipulated during carcinogenesis, such as apoptosis, chemoresistance, angiogenesis, metastasis, cell-cycle progression, proliferation, and invasion. For instance, the biological process of apoptosis relies on a delicate equilibrium between pro- and anti-apoptotic factors to determine the fate of cells [8]. Intriguingly, it has been shown that AS generates opposing regulators of apoptosis, suggesting that AS plays a critical part in a cell’s life-or-death decision-making [43]. BCL2 Like 1 (BCL2L1) is one of the many regulators of apoptosis and a member of the BCL2 Apoptosis Regulator (BCL2) family [8,44]. Alternative splicing of BCL2L1 (BCL-X) results in either a long anti-apoptotic variant (BCL-xl) or a short pro-apoptotic variant (BCL-xs) and this splicing switch is facilitated by the SRSF1 splicing factor [45,46]. High expression of SRSF1 was reported to generate two isoforms of MAPK interacting serine/threonine kinase 2 (MNK2), namely MNK2a and MNK2b, in CRC cells [47].

The high expression of SRSF1 causes an imbalance between the two isoforms with an upregulation of MNK2b and downregulation of MNK2a. Consequently, this inhibits the p38a-MAPK signaling pathway, which results in increased cell proliferation and a decreased rate of apoptosis [47]. Although high expression of the variants contributing to the imbalance in apoptotic factors is reported to play a role in the development and progression of different types of cancers, the specific mechanisms are not yet fully understood [47,48,49]. Table 2 shows some of the splicing events and genes that undergo AS that are involved in pre-mRNA splicing in CRC. These include genes such as RNA-binding proteins (RBP) and their alternatively spliced variants/isoforms resulting from the dysregulation of splicing regulators.

## 6. Contribution of PRMTs and SFKs Regulatory Networks in CRC Carcinogenesis

### 6.1. The Role of Alternatively Spliced Transcripts of PRMTs in Colorectal Cancer

**Figure 4:**
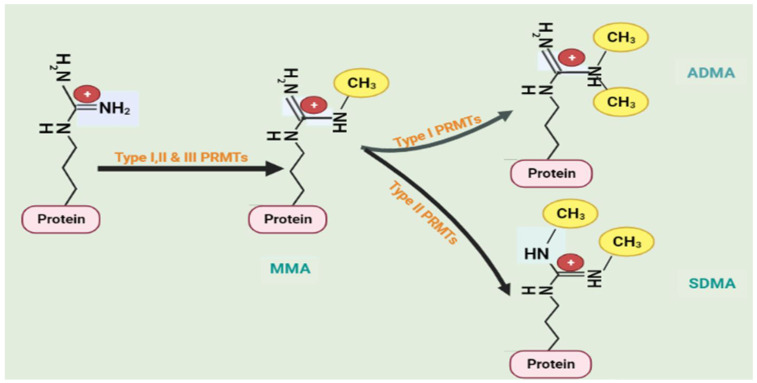
Three forms of protein arginine methyltransferases (PRMT) methylate particular arginine residues: Type I PRMTs (PRMT1-4, PRMT6, and PRMT8) catalyze the asymmetric dimethylarginine (ADMA); Type II PRMTs (PRMT5 and PRMT9) catalyze the symmetric dimethylarginine (SDMA); and Type III PRMT (PRMT7) produces a single methyl group to the single side of nitrogen of arginine residue (MMA). Created with BioRender.com (accessed on 8 June 2023).

Regardless of the current knowledge regarding the contribution of AS events/variants to the development of cancers, the contribution of AS in CRC remains understudied. This is particularly true when it comes to identifying AS events in splicing regulators such as PRMTs and SFKs and their contributions to CRC. PRMTs are a group of enzymes that catalyze arginine methylation, which is currently recognized as a widespread post-transcriptional modification in many proteins [76]. The pivotal role played by arginine methylation in mammals is well recognized and includes, but is not limited to, splicing regulation, RNA metabolism, DNA damage repair, phase separation, and signal transduction [77]. There are three different classes of arginine methyltransferases (PRMT I, PRMT II, and PRMT III) that have been identified based on the final end product (when the methyl group is bonded to the R residue. The formation of monomethylarginine (MMA) is the initial product for all classes of PRMTs [78]. The subsequent methylation process varies between enzyme classes. PRMTs 1, 2, 3, 4, 6, and 8 are class I arginine methyltransferases, which catalyze the conversion of MMA into asymmetric demethylated arginine (ADMA) [78,79]. Unlike type I PRMTs, PRMT5 and PRMT9 are class II arginine methyltransferases, which further catalyze MMA conversion into symmetrically dimethylated arginine (SDMA), whilst PRMT7 is the only enzyme in the group of class III arginine methyltransferases, and functions to catalyze the production of MMA [78,79]. This process is illustrated in Figure 4.

**Figure 5:**
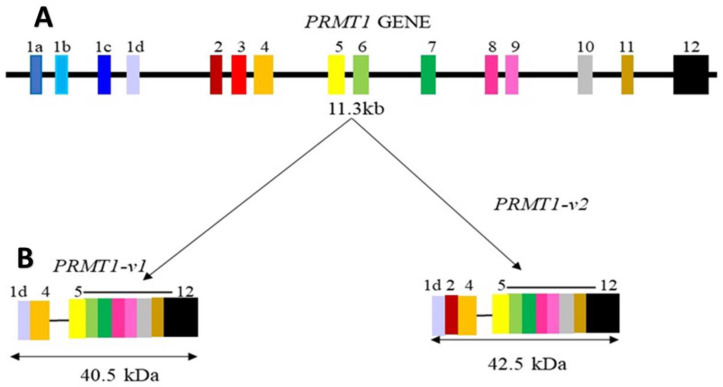
The PRMT1 gene’s genomic context and the structure of its protein products. (**A**) The PRMT1 gene spans 11.3 kb and has 12 constitutive exons, with exon 1 being subdivided into 4 alternative exons (exon 1a–1d). (**B**) The exon composition of PRMT1-v1 and PRMT1-v2. The vertical lines depict the sequences of intron boundaries. Each protein isoform’s molecular weight is presented in kilodaltons (kDa).

Since PRMTs play an important role in arginine methylation, PRMTs are involved in the same processes that require arginine methylation, including the transcriptional and post-transcriptional regulation of gene expression, DNA damage repair, cell-cycle check-points, mRNA processing and translation, as well as intracellular signaling during development and disease progression, particularly in cancers [9,11,78,79]. Over the past decades, studies have shown dysregulation of PRMTs to be associated with cancer progression and metastasis in mammals but the full scope on how the alternatively spliced PRMTs (PRMT isoforms) play a role in tumorigenesis is not yet clearly understood. A study by Adamopoulos et al., 2019 identified a number of AS events that resulted in multiple PRMT1 transcripts which are predicted to encode new protein isoforms [80]. Amongst the pool of PRMT1 variants, two splice variants of PRMT1 (variants v.1 and v.2) (shown in Figure 5) were reported to be significantly upregulated in CRC and their overexpression was associated with the nodal status and histological grade of tumors in colon cancer [80,81,82,83].

The AS event *PRMT1-51042-ES,* reported to be highly expressed by cytotoxic T-helper cells, was identified as an independent predictor of overall survival, genomic instability, and poor prognosis in CRC [84]. PRMT1∆arm, a variant of PRMT1, is missing exons crucial for organizing the dimerization domain necessary for enzymatic activity. As a result, PRMT1∆arm is unable to methylate arginines, but retains the chromatin-binding capacity, competitively limiting the binding of active PRMT1 and ultimately leading to increased chances of malignancy [85]. Given these findings, PRMT1 variants and the AS events leading to these variants may serve as useful prognostic, diagnostic, and/or therapeutic biomarkers for CRC. However, further studies are needed to determine if other types of PRMTs may elicit the same effect in CRCs.

Alterations in splicing factor expression appear to be a significant cause of aberrant splicing profiles, although the processes behind this shift in splicing factor expression in tumors remain poorly understood. Apart from PRMTs, a group of enzymes known as splicing factor kinases (SFKs), which play a role in AS, have been investigated [85]. Serine/arginine protein kinase 1 (SRPK1) is reported to play an important part in AS regulation through phosphorylation of different splicing factors rich in serine/arginine domains (SR proteins), including serine/arginine rich splicing factor 1 (SRSF1) [86,87,88]. Similar to PRMTs, SRPK1 is reported to be overexpressed in many types of malignancies, including CRC. The expression levels of SRPK1 were associated with clinical factors such as TNM staging, and poor disease prognosis or outcome [89,90,91,92,93]. The proper regulation of SRPK1 is crucial in the maintenance of normal physiologic and pathological states in eukaryotic cells, including splice site selection, mRNA export, spliceosome assembly, and translation [94].

### 6.2. The Role of VEGF in CRC and Metastasis

**Figure 6:**
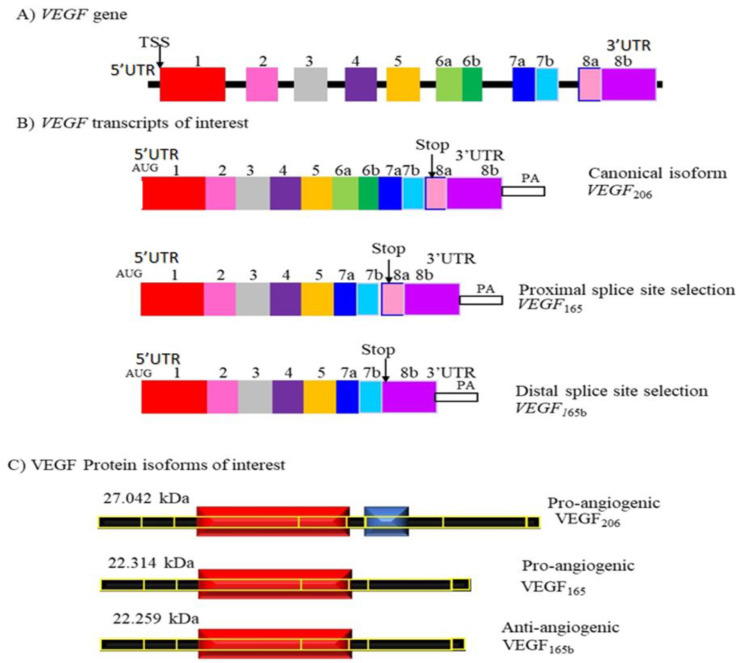
The VEGF-A gene. (**A**) Genomic structure of VEGF-A gene. Transcription starts at TSS. (**B**) VEGF transcripts of interest. VEGFxxx and VEGFxxb isoforms are produced via terminal exon alternative splicing of the VEGFa gene. AUG, translation start; UTR, untranslated region; PA, polyA tail. (**C**) VEGF_165_ and VEGF_165_b protein structures as two major isoforms of the family. The protein’s amino acid number (xxx) names the isoforms.

Vascular endothelial growth factor (VEGF) is a multifunctional cytokine that is involved in angiogenesis through the binding and activation of receptors (VEGFR 1 and 2) on endothelial cells [95,96]. VEGF can undergo alternative splicing to form various isoforms. In particular two of these isoforms, VEGF_165_b and VEGF_165_ are formed via the selection of the proximal splice site (SPP) and distal splice site (DSS) in the terminal of exon 8, as shown in Figure 6 [95].

Dysregulation of SRPK1 is believed to play a role in the splicing switch from the VEGF_165_b to the VEGF_165_ isoform. VEGF_165_ has been shown to promote cell growth and migration [53]. SRPK1 facilitates the splicing switch of VEGF_165_b to the VEGF_165_ isoform by phosphorylating the splicing factor (SRSF1) and promoting proximal splice site usage, ultimately leading to the increased expression of the proangiogenic VEGF_165_ isoform [96]. Furthermore, the dysregulated expression of SRPK1 in breast cancer increases the phosphorylation of RNA-binding motif protein 4 (RBM4). This leads to the production of RBM4-specific splicing variants of myeloid cell leukemia 1 (MCL-1) and Insulin receptor (IR). The isoforms MCL-1s and IR-B lead to decreased or inhibited apoptosis of malignant cells [54]. Although there is limited knowledge of the specific AS events/variants of SRPK1, the current evidence is clear that dysregulation of SRPK1 affects the phosphorylation of splicing factors, eventually contributing to angiogenesis and tumorigenesis. Hence the available literature suggests that SRPK1 has the potential to function as either an oncogene or tumor-suppressor gene.

## 7. Therapeutic Potential of Splicing Disrupter Drugs in Cancer and Colorectal Cancer

Large-scale genomic studies concentrating on single-cell RNA sequencing and characterization have proven to be powerful methods to establish how protein-coding and non-coding RNA transcription and processing are dysregulated in numerous malignancies, thus providing insight into the variety and complexity of tumors [97]. In recent years, substantial evidence gathered thus far suggests that the identification of cancer-specific AS variations has the potential to provide novel therapeutic targets in cancer patients [11,97,98]. Different therapeutic strategies have been used to target the complex mechanisms of AS, while another focus has been on the use of whole transcriptome sequencing to identify novel therapeutic targets. Some therapeutic strategies include targeting trans-regulatory factors of splicing, including the spliceosome complex and splicing regulating factors. Another strategy involves the use of splice-switching oligonucleotides (SSOs), which have been used to correct aberrant AS or induce the expression of a splice variant, and another option is the targeting of a novel CRC-relevant splice variant for therapeutic purposes [98,99,100].

Post-transcriptional modification remains the most important process associated with spliceosome functions, and efforts have been made in developing splicing disrupter drugs/inhibitors that specifically target PTM [97]. Dysregulation of PTM can alter the function of splicing factors and several compounds that have the ability to inhibit different modifications, altering PTM. These include inhibitors of CLKs (CDC-like kinases), SRPKs, and PRMTs, which have been screened, with some showing promise as anti-cancer drugs [100]. One important PTM is methylation by PRMT5, a type II PRMT which is critical in the recruitment and assembly of spliceosome components [37]. The inhibition of PRMT5 as well as PRMT1 and CARM1 have been shown to cause splicing inhibition and display anti-cancer properties in several cancers [101].

A vast number of patents have been filed for PRMT inhibitors from both academic laboratories and the pharmaceutical industry [102]. JNJ-64619178 and GSK3326595 are some examples of PRMT5 inhibitors that are reported to be in human phase-1 clinical trials in patients with advanced or recurrent solid tumors [103]. The treatment of THP-1 cells, a leukemia monocytic cell line with a specific PRMT5 inhibitor (EPZ015666), was reported to decrease levels of SDMA methylation and affect cell proliferation negatively [101]. It is worth noting that these inhibitors can also be used in combination with other drugs/inhibitors. For example, GSK3326595 was used together with anti-PD1 therapy in hepatocellular carcinoma (HCC) and improved efficacy was noticed, suggesting that this combination might be worth testing in future HCC clinical trials [104]. However, the presence of multiple PRMT isoforms with distinct functions can cause interpretation to become difficult, as such PRMT inhibitors must exhibit isoform specificity, being able to target one isoform enzyme. Non-specific global inhibitors of methyltransferase cannot be used to precisely target isoforms or be used in studies to elucidate the function of various isoforms. The desirable solution to this is the development of a potent and isoform-selective small-molecule inhibitor.

PRMT1, -3, -4, -6, -7, and -8 possess a region known as cavity-2, beneath the dimerization arm. This region is responsible for dimerization and the activity of these PRMTs [105]. The sequences of amino acids lining the cavity differ amongst different PRMTs as well as different isoforms. The differences of the residue sequences amongst different isoforms may allow for the specific targeting of different isoforms [106]. Isoforms also complicate the assessment of the efficiency of inhibitors. This is normally assessed through IC 50 values or the inhibition constant K [107]. An example of one class of these small inhibitors is the ethanediamine-heterocycle compounds. These compounds appear to selectively inhibit different PRMTs and different PRMT isoforms, with different members of this class being able to act as a pan-PRMT inhibitor or be selective for only one isoform [108].

In patients with melanoma, GSK3326595 in combination with Palbociclib (CDK4/6) inhibitor may assist in decreasing the chances of drug resistance [37]. In contrast to PRMT5, PRMT1 catalyzes ADMA and is overexpressed in multiple cancers [37,38]. Overexpression of PRMT1 reduces the expression of RBM15, which is reported to play a role in hematopoiesis and subsequently affects megakaryocyte terminal differentiation [103]. A completed phase I clinical trial with PRMT1 (GSK3368715) inhibitor was reported to inhibit cancer cell growth in patients with advanced solid tumors and diffuse large B cell lymphoma (DLBCL) [97]. A pre-clinical study using a combination of inhibitors of PRMT1 (MS023) and PRMT5 (EPZ015666) demonstrated an efficient anti-cancer effect in lung cancer and pancreatic cancer cell lines [109]. Currently, there are two CLK (SM08502 and CTX-712) inhibitors that are available for oral consumption which were reported to be in phase I clinical trials in the year 2020 [110]. These inhibitors are reported to target the phosphorylation process of SRSF6 and enlarge nuclear speckle, and have shown great potential to move to phase II clinical trials [110]. Other inhibitors showing great potential are two SRPK (NCT04247256 and NCT04652206) inhibitors, which are reported to be in phase II clinical trials [110]. These inhibitors are administered together with docetaxel and have shown anti-tumour activities in triple-negative breast cancer cells. Currently, there are limited reports on inhibitors in clinical trials that are specific to CRC. Given the data on targeting cancer through PTM inhibition, there is limited data on using splicing disrupter drugs/inhibitors in colorectal cancer. Exploiting the alternative splicing machinery may help in understanding the downstream pathways regulated by PRMTs and splicing factors. This may lead to the discovery of novel opportunities that can be used to exploit the vulnerability of colorectal cancer to splicing inhibitors. These new therapeutic strategies offer great potential to treat CRC, most particularly in low- and middle-income countries.

## 8. Limitations and Challenges of Using AS in a Clinical Setting

It is clear that dysregulation of PTM, such as overexpression of splicing regulators, plays a role in tumorigenesis and progression, but many challenges and questions still remain. Most of the present efforts are directed at determining which of the detected splicing changes are important to the diseases and how those splicing changes may be utilized to inform the development of new therapies and the refinement of existing ones. While there is still a gap in our knowledge, there has been substantial progress made and exciting knowledge gathered in recent years.

Targets that undergo altered splicing in cancer can now be more easily identified because of the development of high-throughput screening (HTS) tools that span whole-genome and exome sequencing. Most research has generated useful primary insights, but we still lack the data needed to further the development of molecular therapies. A minimum of 60 million reads is generally considered as a minimum requirement for accurate splicing quantification when analyzing RNA sequencing data [111,112]. Although there are statistical tools created to quantify alternative splicing variants, such as rMATS, sQTLS, and LeafCutter, choosing the right analysis parameters and experimental design is critical [113,114,115]. Generally, the lack of standardized pipelines for identifying and quantifying alternative splicing events accurately and reliably remains a technical challenge. Although various experimental techniques, such as RNA sequencing, microarrays, and RT-PCR, can detect alternative splicing events, there is a lack of standardized protocols and computational tools for their analysis. This can introduce variability and hinder the translation of alternative splicing into clinical practice. Methods such as RT-PCR, which is a traditional molecular biology technique, have a limited throughput and are usually time-consuming. Although RNA sequencing is a HTS method, this method can be very expensive and require sophisticated bioinformatics analysis and this is generally a disadvantage in several African countries.

Despite the fact that Africa is home to around 15% of the world’s population, it is estimated that just 2% of all clinical trials ever undertaken take place on the continent [113]. A survey of the National Institutes of Health trial repository ClinicalTrials.gov reveals that there have been 736 clinical trials carried out throughout Africa [113]. Of these, only 26 were interventional studies linked to cancer, and only six of these trials were carried out in nations with predominately Black patients [113]. Despite the fact that it is common knowledge that research-based solutions have the potential to have a significant influence on the region’s high death rates, African nations remain under-represented in cancer research. Some of the reasons for this include lack of research resources (research and development funding, and infrastructure and technology), collaboration and partnerships, and health system sustainability. Addressing the finance-related challenges in cancer drug discoveries in African countries requires a multifaceted approach. Increased international collaboration, public–private partnerships, and innovative funding mechanisms could facilitate research and development efforts. Furthermore, governments and international organizations can work together to prioritize and allocate more resources towards cancer research and healthcare infrastructure, thereby enhancing the overall landscape of cancer care in Africa. Ultimately, fostering a supportive financial environment will play a pivotal role in making essential cancer drugs more accessible and advancing the fight against cancer in the region.

Another minimum requirement in the area is reporting only splicing events with a difference of 10–20% across samples. As this threshold represents the upper boundary of reliable detection and validation in orthogonal assays, it is often reported as a noteworthy change; however, this does not always equate to functional relevance [112,114,115]. Additionally, the accuracy at which intron retention is assessed remains a challenge [97]. Given that the choice of standard requirements (analysis pipelines and detection thresholds) differs across studies, this can dramatically impact the conclusions made from the studies. The identification of crucial cancer-specific splicing events and variants among hundreds of splicing alterations, which are simply the result of mutations or altered expression of splicing factors and are not directly connected with the illness, is another significant and vital difficulty to overcome. It may be difficult to define and isolate appropriate control samples, which might be a barrier to determining whether or not these events occurred.

The complexities of CRC are frequently associated with a number of biomarkers and phenotypes, making the discovery of molecular therapeutics difficult. While alternative splicing has been extensively studied, the functional consequences of specific splicing events are often poorly understood. As a result, knowing how splicing may drive or shape cancer, as well as recognizing and defining splicing dysregulation in cancer, is critical for disease diagnosis and treatment. Additionally, determining the impact of particular splicing events in the setting of cancer is challenging. Furthermore, the degree of splicing modifications varies between clinical patients and malignancies, making it challenging to pinpoint a suitable splicing event for therapeutic correction. The current review highlighted certain splicing events that have been linked to cancer, making it more possible to rectify aberrant alternative splicing using splicing disrupter drugs/inhibitors.

## 9. Conclusions and Future Perspectives

**Figure 7:**
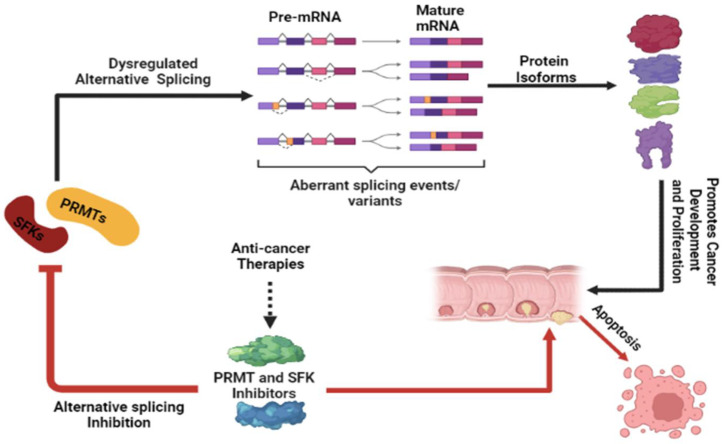
Therapeutic potential of PRMT and SFK inhibitors: The alternatively spliced PRMTs and SFKs expressed as a result of dysregulated AS generate aberrant splicing events/variants that are translated into protein isoforms that are oncogenic. The aberrantly spliced events/variants can be inhibited by splicing disrupter drugs that have the potential to be used as anti-cancer therapies. To enhance the effectiveness of PRMT and SFK inhibitors, they can be used together with other anti-cancer therapies. Created with BioRender.com (accessed on 13 June 2023).

This review article discussed the use of treatments and diagnostic tools based on AS in the control and management of colorectal cancer. Several studies have explored the possibility of creating small-molecule medicines or inhibitors that can specifically target highly structured elements in disease-causing mRNAs that have undergone aberrant processing [116]. The ability of small molecules to bind certain structural conformations inside introns and elicit structural alterations that influence alternative RNA splicing and gene expression has been established [117]. The therapeutic potential of splicing disrupter drugs (PRMT and SFK inhibitors) that may be used to target aberrant AS in CRC is demonstrated in Figure 7.

Despite growing evidence that faulty splicing regulation contributes to carcinogenesis, the precise function of splicing in cancer pathogenesis, especially in colorectal malignancies, remains unclear. However, specificity and delivery efficiency are among the key hurdles being faced by scientists. Targeting splicing might bring about fresh and appealing therapeutic strategies for treating cancer. Insights into splicing dysregulation in solid tumors and the development of more effective RNA-based anti-tumor therapies may emerge from ongoing clinical investigations. Targeted therapeutics will benefit substantially from a systematic assessment of the functional activities of RNA isoforms that are unique to tumors. As we learn more about the effects of splicing dysregulation in human malignancies, we find that many splicing alterations are tissue- and cell-specific. Splicing regulators’ roles in normal tissue and the consequences of their dysregulation in the setting of cancer need to be dissected with sufficient precision at the large-scale genomic level. Cancer-associated splicing regulators are important for identifying novel biomarkers and establishing novel approaches to therapy, but our current understanding of their cell-type specificity and roles is limited. Despite the fact that the area is still in its early stages of development and despite the fact that there are challenges with precision and delivery, therapeutic approaches that target cancer-specific AS variants are an exciting and novel strategy for preclinical and clinical research, with the potential for considerable clinical impacts.

## Figures and Tables

**Table 1:** Aberrantly spliced events that may be used as diagnostic biomarkers in different types of cancers.

Gene(s) of Interest	Splicing Regulator	Spliced Events	Type of Marker	Description	Ref.
CCND1	SRSF1	G/A polymorphism at exon 4 and intron 4	Prognostic	Upregulated and promotes cell-cycle and cell proliferation via tumour suppressor protein Rb	[9]
VCL, TPM1, and CALD1	SF3B4	45,439 AS events, predominantly exon Skipping event	Prognostic	Upregulated and associated with patient survival.	[24,26]
CDK10, TP53, MAP4K3, and ERBB2IP	DNA-directed RNA polymerase II and the RNA spliceosomal complex	2589 alternative splicing events where ES occurrences predominated as the most common	Prognostic	Contribute to the development and spread of cancer	[27]
MAPKBP1	N/I	60,754 AS events. ES was the most predominant AS event	Prognostic	Six mRNA splice variant prognostic models were significantly associated with the OS.	[28]
PAR3 and NUMB	TDP43/SRSF3 complex	A total of 45,421 splice events were detected.	prognosis, relapse, and metastasis	Upregulation of TDP43 associated with poor prognosis	[29]
NFIC/CTF5	MCPIP1	A total of 762 AS events were detected	cell cycle progression and proliferation	Increased levels of MCPIP1 were correlated with prolonged OS	[30]
TET3, FGFR2, p120-Catenin and CD44	ESRP1	Exon 2 and 3 skipping in p120-catenin. Cassette exon in CD44	Diagnostic	Upregulated and plays a central role in epithelial to mesenchymal transition. Increased expression of CD44s.	[31,32,33,34]

**Table 2:** AS events involved in cancer-promoting processes in CRC.

Gene	Splicing Event	Variant/ Isoform	Biological Function	Type of Cancer	Ref.
**Angiogenesis** 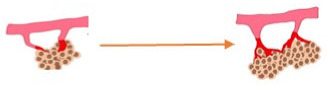					
VEGFA	Alternative 3′ splice site in exon 8	VEGF_165_	promote cell growth and migration, proangiogenic	Colorectal, skin, prostate, and renal cancer	[11,50,51,52]
VEGFR	Intron 13 retention	mVEGFR-2	Proangiogenic and lymphangiogenic	Colon cancer, colorectal neoplasms	[53,54,55]
**Apoptosis** 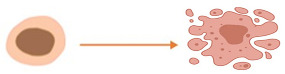					
MNK2	Inclusion of exon 14b and skipping of exon 14a	MNK2b	Increased cell-growth and decreased cellular apoptosis (Pro-oncogenic)	Colorectal, breast, and lung cancer	[11,46,55,56]
BCL2L1	5′ alternative splice site usage in exon 2	BCL-xl	Inhibits- or Anti-apoptotic	Colorectal, lymphoma, prostate, and breast cancer	[37,43,57]
PKM	Skipping of exon 9 and inclusion of exon 10	PKM2	Cell-proliferation, tumorigenesis, and anti-apoptotic	Colon, ovarian, gastric, and liver cancer	[45,58,59,60]
MRPL33	Inclusion of alternative exon 3	MRPL33-fl	Promotes cell-growth and anti-apoptotic	Gastric and colon cancers	[61,62]
**Proliferation** 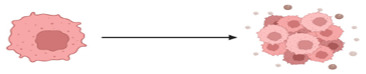					
MNK2	Inclusion of exon 14b and skipping of exon 14a	MNK2b	Increase cell-growth and proliferation	Colorectal, breast, and lung cancers	[47,56]
CD44	Contains exon v4-10	CD44v4-10	Tumour-cell proliferation	Colon and intestinal cancer	[46,47,61,62,63]
PKM	Skipping of exon 9 and inclusion of exon 10	PKM2	Increase Cell-proliferation	Colon, ovarian, gastric, and liver cancer	[56,57,64]
DBF4B	Exon 6 retention	BDF4B-FL	Increase cell-proliferation and tumorigenesis	Colon cancer	[65]
**Invasion and Metastasis** 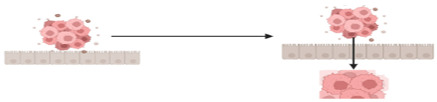					
CD44	Inclusion of variable exon 6	CD44v6	Induces cell migration and promotes metastasis	Colon cancer	[66,67,68]
CAECAM1	Inclusion of exon 7	CEACAM1	Promotes invasion and migration (colon–liver metastasis). Accelerates metastasis and progression	Colorectal cancer, Metastatic melanoma	[69,70,71,72]
MST1R	Exon 11 skipping	RONΔex11	Initiates tumour-cell motility and invasion	Ovarian, colon, lung, and gastric cancers	[73,74,75]

## Data Availability

The data can be shared up on request.
